# On the phenomenon of large photoluminescence red shift in GaN nanoparticles

**DOI:** 10.1186/1556-276X-8-342

**Published:** 2013-07-31

**Authors:** Ahmed Ben Slimane, Adel Najar, Rami Elafandy, Damián P San-Román-Alerigi, Dalaver Anjum, Tien Khee Ng, Boon S Ooi

**Affiliations:** 1 Photonics Laboratory, King Abdullah University of Science and Technology (KAUST), Thuwal 23955-6900, Saudi Arabia; 2 Advanced Nanofabrication and Imaging Core-Lab, King Abdullah University of Science and Technology (KAUST), Thuwal 23955-6900, Saudi Arabia

**Keywords:** Gallium nitride nanoparticles, Ultraviolet electroless etching, Large tunable emission, Photoluminescence red shift, Potential fluctuation, Surface state effect

## Abstract

We report on the observation of broad photoluminescence wavelength tunability from n-type gallium nitride nanoparticles (GaN NPs) fabricated using the ultraviolet metal-assisted electroless etching method. Transmission and scanning electron microscopy measurements performed on the nanoparticles revealed large size dispersion ranging from 10 to 100 nm. Nanoparticles with broad tunable emission wavelength from 362 to 440 nm have been achieved by exciting the samples using the excitation power-dependent method. We attribute this large wavelength tunability to the localized potential fluctuations present within the GaN matrix and to vacancy-related surface states. Our results show that GaN NPs fabricated using this technique are promising for tunable-color-temperature white light-emitting diode applications.

## Background

Optical properties of GaN nanostructures are of great current interest because of the potential application in solid-state lighting [1,2]. In n-type GaN, an ultraviolet (UV) peak at approximately 3.42 eV usually dominates the photoluminescence (PL) spectrum [3]. The blue luminescence at 2.7 to 3 eV peak energy has been extensively studied; this peak dominates due to optically active defects and impurities [4]. Although such defects can be destructive in a device, a well-engineered inorganic nanoparticle approach can offer many advantages [5]. Despite enormous efforts in studying the GaN defect-related emissions [4], there is still a research gap in explaining the origins of PL shift with optical power injection [6]. The localized potential fluctuations within the GaN matrix introduced by the Ga vacancies and impurities are considered in explaining the PL shifts [7]. Reshchikov et al. observed a blueshift with increasing power due to the potential fluctuation in bulk p-type GaN [8]. On the other hand, in nanostructures having a large specific area, the surface states effect became significant in influencing the carrier recombination mechanism [9]. However, to our knowledge, a large PL red shift with increasing excitation power was not reported and requires further investigations.

In this paper, we demonstrated the fabrication of a group III nitride-based nanoparticle (NP) using a UV-assisted electroless chemical etching method and explained the switchover in optical emission mechanism from defect-dominated to bulk-dominated PL transitions. The resultant GaN NPs are chemically stable, simple to fabricate, and easy to integrate and, most importantly, offer tunable broadband emission. We studied the emission mechanism of such novel GaN NPs, which showed controllable red shift of approximately 80 nm (approximately 600 meV) with increased optical excitation power. The tunability feature renders these nanoparticles as a good candidate for further development of tunable-color-temperature III-N-phosphor-based white light-emitting diodes (LEDs) which are essential for matching room lighting with human circadian rhythms [10].

## Methods

The substrate used in this study consisted of a 30-μm-thick Si-doped GaN epitaxy grown on *c*-plane (0001) sapphire (α-Al_2_O_3_) substrate with a measured resistivity of less than 0.03 Ω cm. The estimated dislocation density and measured carrier concentration of the film are 1 × 10^8^ cm^−2^ and 2 × 10^18^ cm^−3^, respectively. Prior to wet etching in a HF/CH_3_OH/H_2_O_2_ (2:1:2) solution under UV illumination, 10-nm thin strips of platinum (Pt) were sputtered onto the GaN samples at one end of the surface to complete the loop for electron–hole exchange between semiconductor and electrolyte [11]. The resultant nanostructure layers were later transferred onto a Si wafer at subsequent room temperature and 77 K for PL measurements using Jobin Yvon’s LabRAM ARAMIS microphotoluminescence (μPL) spectroscopy system (HORIBA, Ltd., Minami-ku, Kyoto, Japan). The optical excitation was produced using a helium-cadmium (HeCd) laser emitting at 325 nm with a <10-μm spot size. The scanning and transmission electron microscopy (SEM and TEM) investigations were performed using FEI Quanta 600 and FEI Titan G^2^ 80–300 electron microscopes (FEI Co., Hillsboro, OR, USA), respectively.

## Results and discussion

**Figure 1:**
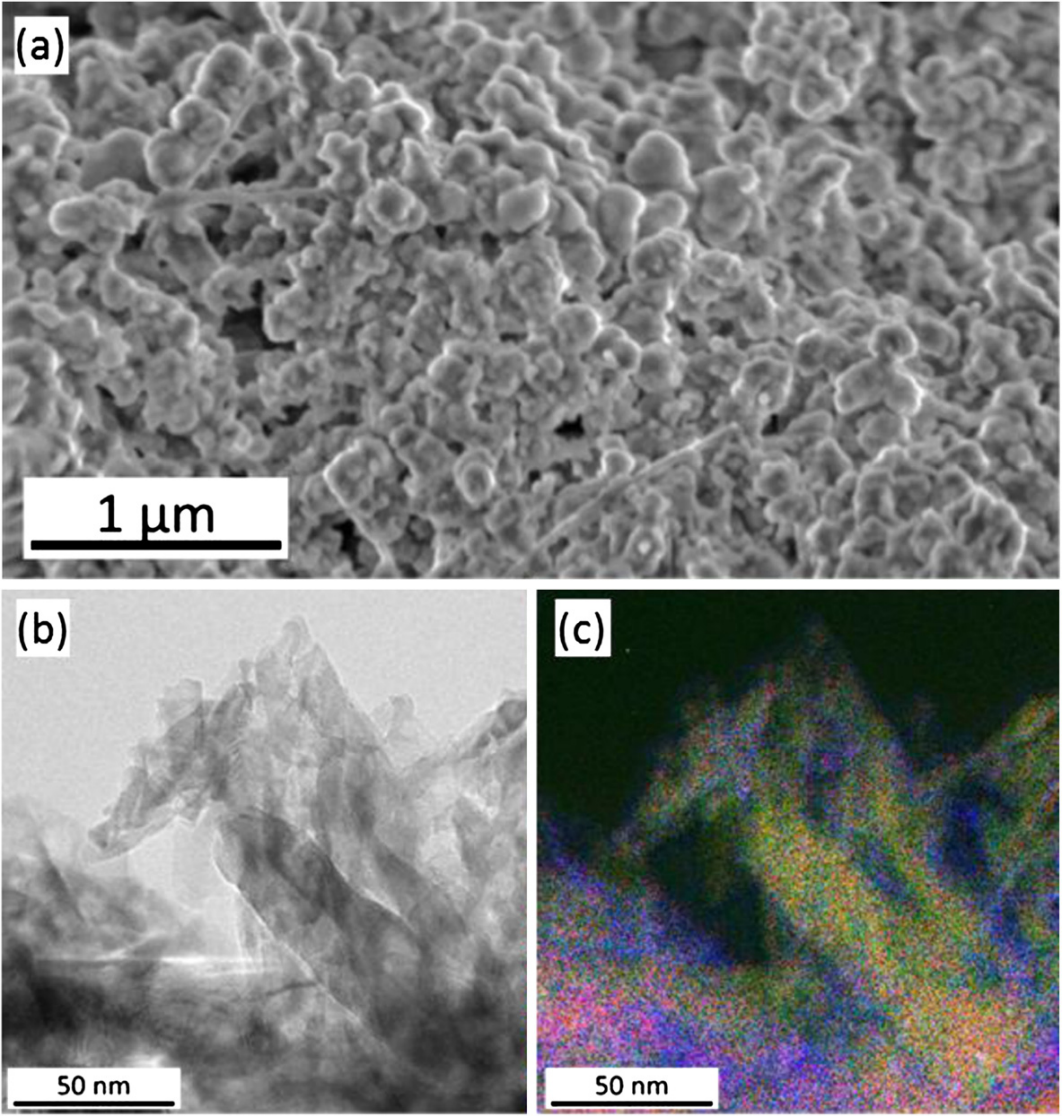
**SEM and TEM images and elemental maps. (a)** SEM image of the NPs prepared using UV metal-assisted electroless etching technique and **(b)** TEM image of NPs. **(c)** Overlaid elemental maps of Ga, N, and O in red, green, and blue, respectively, acquired by EFTEM.

Figure 1a shows the SEM image of the GaN NPs on a Si substrate in a grain-like structure having NPs with sizes ranging from 10 to 100 nm. By high-resolution TEM (Figure 1b), we observed adjoining single-crystal GaN NPs with each particle surrounded by the amorphous-like boundary. The electron energy loss spectroscopy (EELS) analysis revealed the oxygen amount to be about 20 at.%. The spatial distributions of all three constituent elements, namely Ga, N, and O, are determined and acquired using the energy-filtered TEM (EFTEM) technique (see in Figure 1c). It can be noticed from Figure 1c that the O map (blue) is mostly present in the surrounding of NPs which is in agreement with results obtained from EELS. The presence of oxygen in the nanoparticle can be explained by the finite surface oxidation of GaN simply due to the large specific surface.

**Figure 2:**
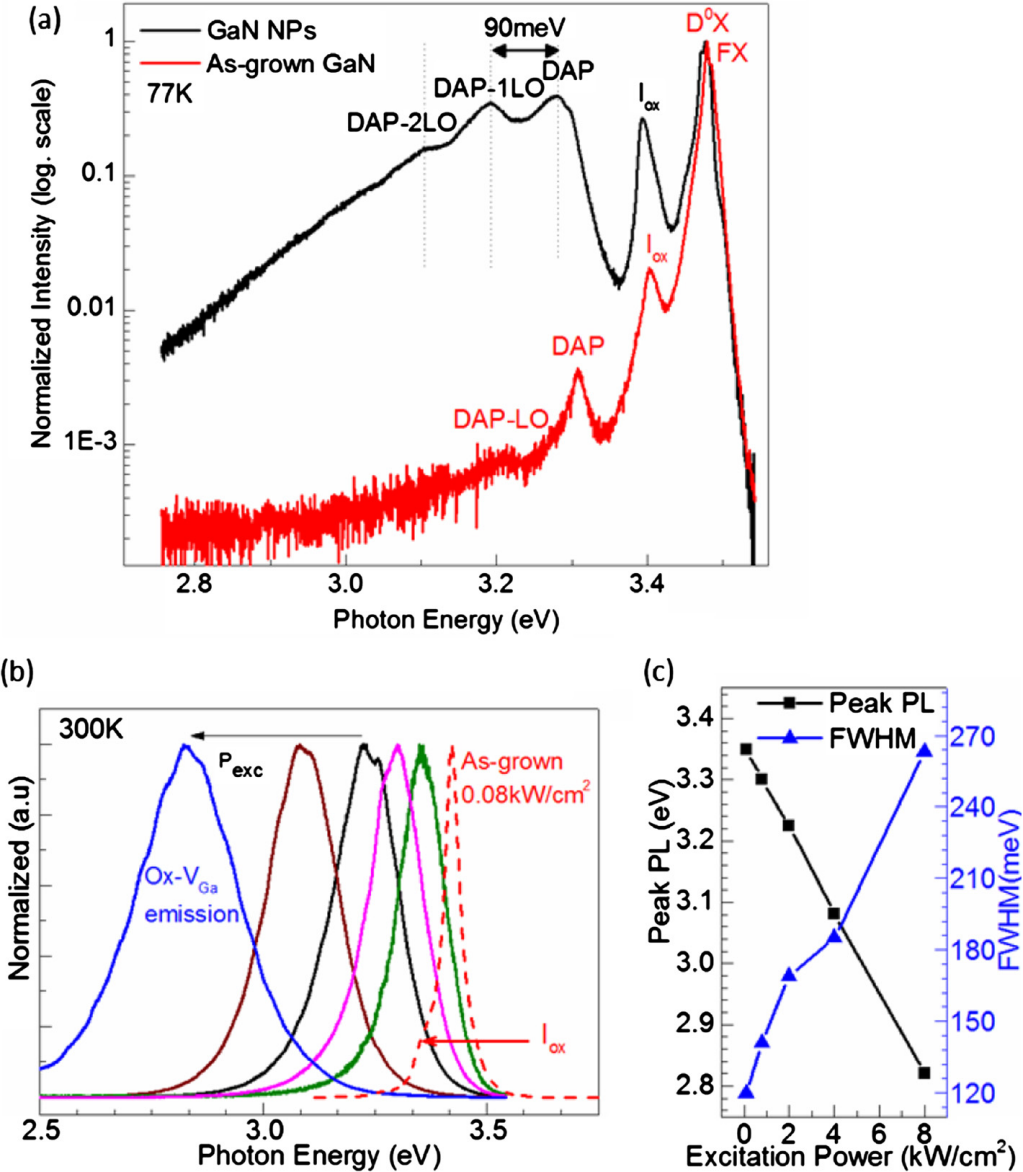
**Emission spectra of GaN epitaxy and GaN NPs, peak PL photon energy and FWHM dependence. (a)** Normalized 77 K μPL emission spectra of as-grown 30-μm GaN epitaxy and GaN NP cluster with semi-log scale. **(b)** Normalized room temperature μPL emission spectra of as-grown GaN (dashed line) and GaN NP (solid line) cluster excited with increasing laser power (0.08, 0.8, 2, 4, and 8 kW/cm^2^). **(c)** The peak PL photon energy (black squares) and the FWHM (blue triangles) dependence over the excitation power.

In order to understand the difference in the emission mechanism of as-grown GaN epitaxy and the as-fabricated NPs, we studied the normalized μPL spectra at 77 K. Figure 2a shows disparate emission characteristics of GaN in both GaN epitaxy and NPs. In the as-grown GaN epitaxy, we clearly observe the existence of one relatively sharp peak at the UV region, 3.479 eV (approximately 356 nm) with a full width at half maximum (FWHM) of 13 meV, which is attributed to the donor-bound exciton peak (*D*^0^*X*) [3]. The small hump at 3.484 eV is assigned to the free-excitonic peak (FX). We attribute the small PL peak *I*_ox_ at 3.4 eV mainly to oxygen impurities that originated from Al_2_O_3_, i.e., the oxygen impurity-related donor-to-valence band transitions as reported by Chung and Gershenzon [12] and Fischer et al. [13]. The donor-acceptor pair (DAP) peak at 3.308 eV has its longitudinal optical (LO) phonon peak at lower photon energy.

The μPL spectrum of the GaN NPs presents approximately 110-meV red shift that could be attributed to the relaxation of the compressive strain [5], but foremost, we observe a relatively strong/prominent increase of the DAP and *I*_ox_ peak intensities. In the n-type GaN DAP transitions, these acceptor-like sites have been reported by a number of authors to originate from Ga vacancies (*V*_Ga_) [14,15]. The GaN NPs underwent chemical etching, thus resulting in an increase of oxygen and vacancy sites at the surface due to the competition between the formation and dissolution of Ga_*x*_O_*y*_ (Figure 1c). This explains the increase in the emission intensity of DAP peaks.

The power-dependent PL measurement was performed on the NPs. Figure 2b shows a typical room temperature μPL emission spectrum of the as-grown GaN excited at 0.08 kW/cm^2^ together with the excitation power-dependent μPL emission spectrum of the GaN NPs. Compared to the 77 K PL, we observe in the room temperature PL of the as-grown sample a quenching of *D*^0^*X* peak while the FX emission became dominant at 3.42 eV (approximately 362 nm). The broadening in the lower photon energy due to the oxygen impurity is still observable whereas the DAP peak disappeared. Most importantly, room temperature PL of GaN NPs excited at 0.08 kW/cm^2^ exhibits a luminescence peak centered at 3.353 eV (369 nm) which is red-shifted by 69 meV compared to the as-grown sample. As the excitation power increases from 0.08 to 8 kW/cm^2^, we observe an approximate linear decrease of the peak PL photon energy with a total span of 530 meV (Figure 2c). We investigated several spots in the as-grown GaN bulk epitaxy, but no shift with increasing excitation power was observed. Besides the red shift, the measured FWHM shows a direct dependence over the excitation power as it increases from 120 meV (approximately 13 nm) at 0.08 kW/cm^2^ to 263 meV (approximately 40 nm) at 8 kW/cm^2^ (Figure 2c). Such a wide FWHM is twice as large as the measured FWHM of the peak from the as-grown GaN bulk epitaxy where the linewidth broadening at the same power density is 42 meV (approximately 4.5 nm). This FWHM widening indicates a contribution of inhomogeneous broadening in the clusters of NPs.

**Figure 3:**
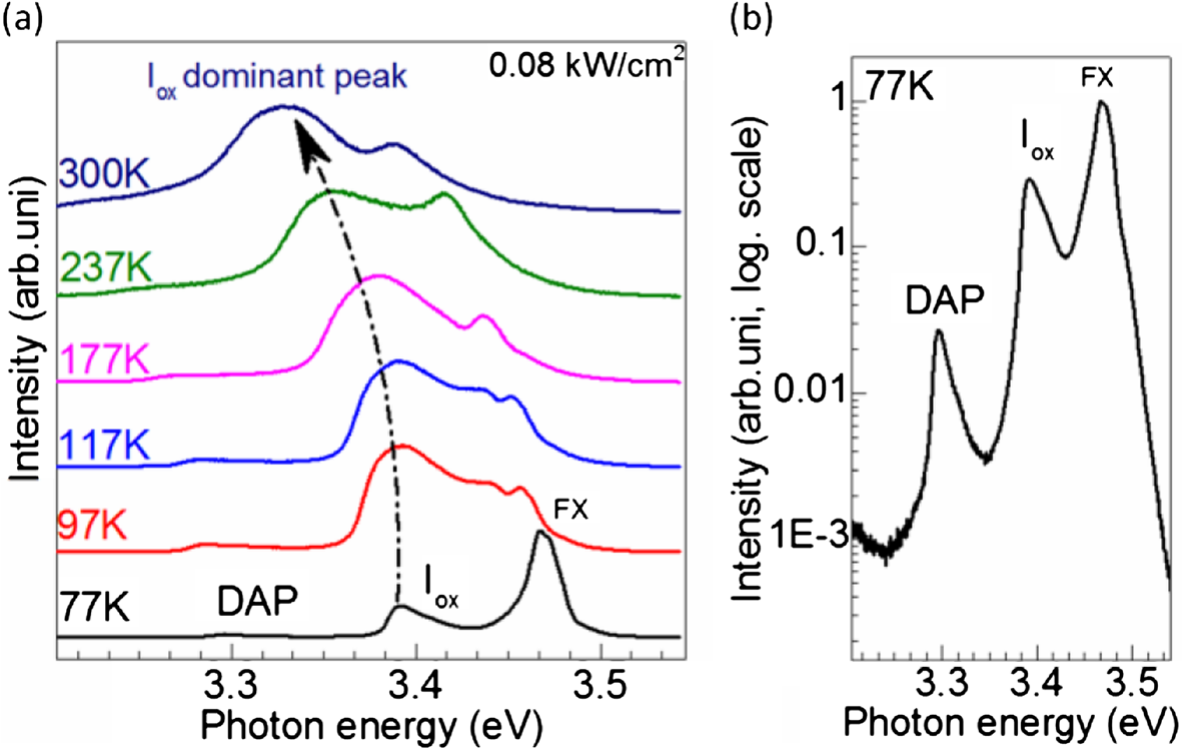
**Temperature-dependent and normalized 77 K μPL emission spectra of GaN NPs. (a)** Temperature-dependent PL of another GaN NPs excited at 0.08 kW/cm^2^. **(b)** Normalized 77 K μPL emission spectrum of GaN NPs cluster with semi-log scale.

For clarity, we turn to another dispersed GaN NPs whose PL spectra are also distinguished with a dominance of the impurity and oxygen-related peaks over the FX peak with increasing temperature (Figure 3a). For comparison, Figure 3b shows the semi-log scale PL of this NP cluster at 77 K, which confirms our previous observation where the DAP and *I*_ox_ peaks increase with respect to those of the as-grown GaN epitaxy (see Figure 2a).

In the following discussion, we investigate the large red shift and linewidth broadening in PL emission of the NPs triggered by the increase of the power density. It is generally accepted that several processes can cause this shift, namely (a) bandgap renormalization [16], (b) changes in the DAP [17], (c) impurity band formation [4], and (d) surface states and/or the potential distribution in the crystal [18,19]:

(a) In bandgap renormalization, the formation of ionization and electron hole plasma leads to the bandgap narrowing [17]. Calculations specific to our material and experimental conditions, based on the empirical relation Δ*E = kn*^1/3^ reported by Lee et al. [16], where *k* is the bandgap renormalization coefficient (*k* ~ 10^−8^ eV cm), *E* is the bandgap energy, and *n* is the carrier density, predict a bandgap narrowing in the order of 20 meV. This prediction is inconsistent with our experimental measurements, specifically considering the large red shift measured, so bandgap renormalization can be safely neglected as a plausible cause.

(b) Due to the Coulomb interaction, transitions related to DAP blueshift with increasing excitation intensity. In fact, the photon energy (*hυ*) is inversely proportional to the distance, *r*, between neutral acceptors and donors, i.e., *hυ* ∝ 1 / *r*. With increasing excitation power, the distance decreases, and as a consequence, the photon energy blueshifts by a few millielectron volt [17]. Conversely, in our case, a significant red shift is observed, and hence, we might ignore the blueshift caused by the Coulomb interaction in these transitions.

(c) The GaN used in this study is n-doped and has a carrier density of 2 × 10^18^ cm^−3^; thus, the red shift might be due to the presence of an impurity band generated from doping concentrations [4].

(d) The potential fluctuations model, on the other hand, explains this large red shift in the PL with increasing excitation power. It is known that the crystalline orientation distortions cause effective bandgap dispersion and thus creates lateral potential fluctuations. Vacancies, impurities, dangling bonds, and strain and structural defects all introduce these fluctuations [18,19]. In our case, the material underwent chemical electroless etching from which a different structural shape and strain in the NPs arises [20]. This coalescence of the NPs induces the formation of boundary dislocations, and additionally, the preferential etching increases the impurity and vacancy defect concentration [20].

**Figure 4:**
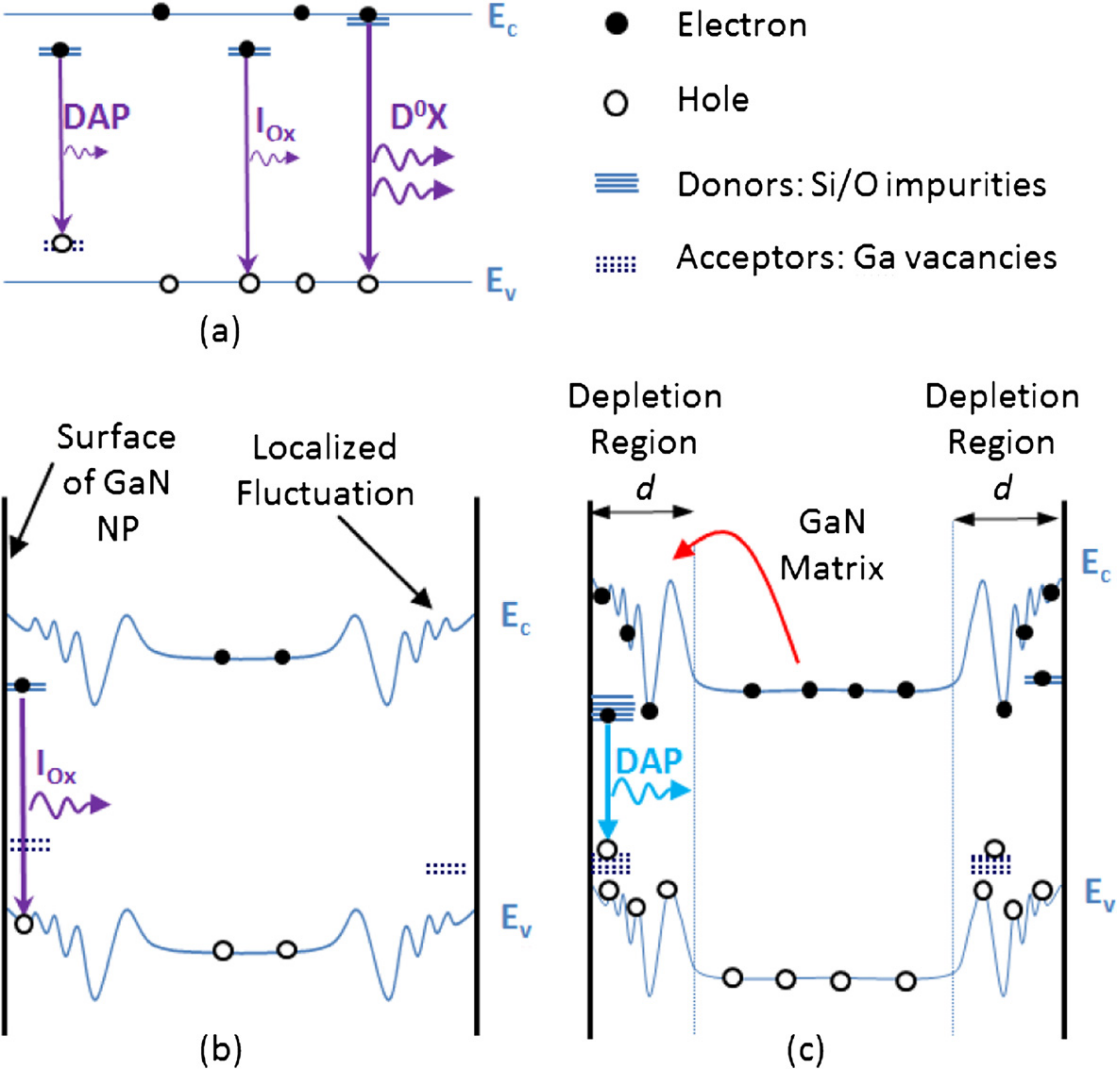
**Schematic representations of potential fluctuation and surface states caused by defects and band distortion. (a)** Bulk GaN. **(b)** NP thoroughly depleted at low excitation power/low temperature. **(c)** NP with high carrier concentration at high excitation power/high temperature has a surface depletion region with small width. Arrows indicate recombination of free electrons and bound holes.

The bandgap dispersion in NPs creates local potential minima where carriers recombine [21] (Figure 4). Upon low excitation power, non-equilibrium electrons and holes are generated and move towards the conduction band minima and valence band maxima, respectively. While in the as-grown GaN, at room temperature, FX transitions are intense. After etching, acceptor-like sites are created in the surface and a small red shift is induced due to the increase of donor-to-valence band and DAP transitions. When we increase the excitation power, more electrons get excited in the conduction band, inducing an electric field screening effect and band flattening in the fluctuated potential bands. As a consequence of these effects, the carrier lifetime is longer and excited carriers have more time to reach lower energy localized states. Electrons overcome the lowered potential barriers (presented by the small red arrow in Figure 4) and get trapped in the deep localized potential minima, where the blue luminescence is stronger. This can be understood if we recall that the wave function of electrons in these local minima is relatively quite spatially extended and thus can easily overlap with the wave function of holes bound in the acceptor-like sites, increasing the probability of such a transition. There may exist many lower energy states and donor trap sites; this recombination would increase the emission linewidth.

Furthermore, due to the extremely high surface-to-volume ratio of the produced NPs paired with localized potential fluctuation, surface states cannot be neglected. The n-type GaN NPs have surface defects; thus, we have band bending in these regions (Figure 4). The creation of surface depletion will change the emission in the GaN NPs. The calculated width of the depletion region in our case is *d* ~ 24 nm, given by [22]$d=\sqrt{2\epsilon_{\mathrm{GaN}}V_{\mathrm{bi}}/qN_{d}},$ where *ϵ*_GaN_ is the static dielectric constant of GaN, *V*_bi_ the potential at the boundary, *q* the electronic charge, and *N*_d_ the donor density. The NP with a width *W* < 2*d* will be totally depleted. *V*_Ga_ centers acting like acceptor sites will be depleted from holes, and FX transitions will dominate. If *W* > 2*d*, both depletion region and non-depletion region can exist. Furthermore, by increasing the excitation power or temperature, the depletion region decreases and the Fermi level increases. Thus, holes populate the acceptor-like sites in the depletion region and electrons populate the donor states; therefore, we have an increase of DAP and donor-like oxygen states and acceptor-like *V*_Ga_ states. This leads to the visible blue emission at higher excitation power. In Figure 4c, the depletion region is a collective representation of trap states due to sharp edges within a NP and across different NPs with size inhomogeneity evident in Figure 1. The sharp edges and/or smaller NP sizes enhance oxidation and therefore increase the density of states and carrier capture cross section of carrier traps, i.e., localized states. In addition, the smaller the NP, the higher the conduction band minima of the local potential fluctuation. The LO phonon enhancement is due to indirect transition from the silicon donor states to the valence band maxima of the local potential fluctuation, which confirms the PL peak broadening.

The emission yield, tenability, and FWHM of our NPs can be modified by controlling the NP size and inhomogeneity. With further process optimization and postprocessing treatments through, for example, annealing and surface passivation, the quality of the quantum yield of the oxide-encapsulated GaN NPs can be improved.

## Conclusions

In summary, GaN nanoparticles with size dispersion between 10 and 100 nm have been fabricated using the UV metal-assisted electroless etching method. A large emission wavelength tunability of approximately 530 meV has been observed from the nanoparticles. We demonstrated that the localized potential fluctuation and surface state effects are responsible for such shift. These fabricated oxide-encapsulated GaN nanoparticles can be used as phosphor for tunable-color-temperature white LED application.

## Competing interests

The authors declare that they have no competing interests.

## Authors’ contributions

ABS carried out the design and the experiment. AN performed the fabrication. DA performed the TEM and related analysis. ABS and TKN analyzed the results and wrote the manuscript. ABS, DPS, and RE drafted the mechanism. BSO conceived of the study and facilitated its coordination. All authors read and approved the final manuscript.
